# Glia-Derived Extracellular Vesicles in Parkinson’s Disease

**DOI:** 10.3390/jcm9061941

**Published:** 2020-06-21

**Authors:** Bianca Marchetti, Loredana Leggio, Francesca L’Episcopo, Silvia Vivarelli, Cataldo Tirolo, Greta Paternò, Carmela Giachino, Salvatore Caniglia, Maria Francesca Serapide, Nunzio Iraci

**Affiliations:** 1 Department of Biomedical and Biotechnological Sciences (BIOMETEC), University of Catania, Torre Biologica, Via S. Sofia 97, 95125 Catania, Italy; loredanaleggio@unict.it (L.L.); silvia.vivarelli7@gmail.com (S.V.); greta.paterno.gp@gmail.com (G.P.); serapide@unict.it (M.F.S.); 2 Neuropharmacology Section, OASI Research Institute-IRCCS, 94018 Troina, Italy; flepiscopo@oasi.en.it (F.L.); ctirolo@oasi.en.it (C.T.); carmelagiachino@libero.it (C.G.); scaniglia@oasi.en.it (S.C.)

**Keywords:** glia, extracellular vesicles, exosomes, cell-to-cell communication, biomarkers, nanotherapeutics, Parkinson’s disease

## Abstract

Glial cells are fundamental players in the central nervous system (CNS) development and homeostasis, both in health and disease states. In Parkinson’s disease (PD), a dysfunctional glia-neuron crosstalk represents a common final pathway contributing to the chronic and progressive death of dopaminergic (DAergic) neurons of the substantia nigra pars compacta (SNpc). Notably, glial cells communicating with each other by an array of molecules, can acquire a “beneficial” or “destructive” phenotype, thereby enhancing neuronal death/vulnerability and/or exerting critical neuroprotective and neuroreparative functions, with mechanisms that are actively investigated. An important way of delivering messenger molecules within this glia-neuron cross-talk consists in the secretion of extracellular vesicles (EVs). EVs are nano-sized membranous particles able to convey a wide range of molecular cargoes in a controlled way, depending on the specific donor cell and the microenvironmental milieu. Given the dual role of glia in PD, glia-derived EVs may deliver molecules carrying various messages for the vulnerable/dysfunctional DAergic neurons. Here, we summarize the state-of-the-art of glial-neuron interactions and glia-derived EVs in PD. Also, EVs have the ability to cross the blood brain barrier (BBB), thus acting both within the CNS and outside, in the periphery. In these regards, this review discloses the emerging applications of EVs, with a special focus on glia-derived EVs as potential carriers of new biomarkers and nanotherapeutics for PD.

## 1. Introduction

Glial cells represent more than 50% of the cells in the central nervous system (CNS), of which astrocytes are the most abundant cell type. Astrocytes are functionally indispensable for normal brain activities as they play a critical function in brain homeostasis [1,2,3]. Alongside their recognized supportive role to their neuronal counterparts, astrocytes regulate synapse formation, they induce the development and plasticity of neurons, contribute to axonal growth and blood-brain-barrier (BBB) formation, and promote neuroprotection thanks to the release of a wide variety of molecules, endowed with neurotrophic, anti-inflammatory and anti-oxidant properties, via a bidirectional astrocyte-neuron crosstalk [3,4,5,6,7,8,9,10,11,12]. Microglia, defined as the resident immune cells of the brain [13], consist of highly adaptable and dynamic cells, recognized to play important roles during development in the healthy CNS [14,15]. In basal, non-activated conditions, “resting” microglia show elongated cell bodies with ramified processes that continuously monitor the local brain microenvironment and interact with neurons, astrocytes and blood vessels [16,17,18,19]. However, both astrocytes and microglia can turn into a very destructive phenotype upon injury, as observed when nigrostriatal dopaminergic (DAergic) neurons degenerate in Parkinson’s disease (PD).

PD is the most common age-dependent movement disorder and the second most prevalent neurodegenerative disease (ND) [20,21,22]. A first characteristic feature of PD is the selective and progressive loss of midbrain DAergic neurons of the substantia nigra pars compacta (SNpc), and their terminals in the striatum, responsible for the gradual impairment of motor function leading to the classical motor features of PD (i.e., bradykinesia, rest tremor, rigidity and postural instability). The second pathologic feature is the presence of cytoplasmic inclusions, called Lewy bodies (i.e., eosinophilic intracellular inclusions composed of amyloid-like fibers and α-synuclein), and dystrophic neurites, called Lewy neurites, in the SN and other areas of the brain [23,24]. Recently, new α- and γ-synuclein immunopathological lesions were reported in human brain [25], raising the possibility that oxidation and/or aggregation of these proteins might be involved in the pathogenesis of some NDs, including PD [25]. The causes responsible for DAergic neuron degeneration still remain unclear albeit compelling evidence clearly implicates a dysfunctional interaction between a number of genes and several environmental factors, especially aging, neurotoxin exposure and inflammation [8,26,27,28,29,30,31,32,33,34,35,36].

Indeed, from the early studies of McGeer and collaborators [37], and during the last three decades, an increasing body of evidence documented in epidemiological, post-mortem, and animal studies, points to astrocytes and microglia as the key mediators of the harmful events leading to the progressive demise of midbrain DAergic neurons in PD [9,26,29,37,38,39,40,41,42,43,44,45,46,47,48,49,50,51,52,53,54,55,56,57,58,59]. Accordingly, a long list of preclinical researches demonstrates that anti-inflammatory treatment may be effective to ease PD symptoms [8,9,29,46,47,48,49,50,56,57,58,59,60,61,62,63].

Notably, glial cells communicating with each other by an array of molecules (e.g., neurotransmitters, neuromodulators, neuropeptides, hormones and neuroimmune regulatory molecules) can acquire a “beneficial” or “destructive” phenotype, thereby enhancing or inhibiting neuronal vulnerability against various noxious stimuli, which poses the “to be or not to be inflamed” dilemma [8].

While a body of studies concentrated on the “harmful” effects of astrocyte and microglial reactions to PD injury, accumulating evidence clearly indicates the ability of astrocytes and microglial cells to exert critical neuroprotective and neuroreparative functions, with mechanism(s) and signaling systems triggering a glial “beneficial” phenotype being actively investigated.

Importantly, astrocytes and microglial cells are pivotal in modulating the stem cell niche that promote neurogenesis, including the survival and identity of neural stem/progenitor cell (NSC)-derived DAergic neurons, thereby regulating adult NSC plasticity in neurogenic niches in the PD brain (recently reviewed by Marchetti et al. 2020 [64]).

Within the different intercellular neuron-neuron, glia-glia, neuron-glia and glia-neuron communication routes, extracellular vesicles (EVs) represent a way to effectively convey in a time- and space-controlled manner biomolecular messengers, such as cytokines, enzymes, mRNAs, non-coding RNAs (including microRNAs) [65]. EVs may be secreted by all cell types, including CNS cells (i.e., neurons and glia) [66]. The EVs include all different kinds of vesicles that can be released by cells. They are classified in three main categories, based on their biogenesis: (i) exosomes (30–150 nm), deriving from the endocytic compartment, (ii) microvesicles (50 nm–2 µm) directly released through a shedding mechanism from the plasma membrane, and (iii) apoptotic bodies (>50 nm) [67]. Currently, none of the techniques available is able to isolate a single subtype of EVs and, for that reason, researchers are trying to generate innovative methods for EV purification, based for example on novel class-specific markers [68]. In light of that, an alternative EV classification divides them purely based on the size: small EVs (<200 nm) and medium/large EVs (>200 nm) [69]. The terms EVs or exosomes will be used in this review according to the nomenclature used in the original publication.

EVs are involved in the pathogenesis of many neuroinflammatory and neurodegenerative disorders, including PD [70]. In fact, amongst the known molecular features of PD, a defective endosomal and lysosomal protein trafficking has been widely reported [71,72]. In turn, EVs biogenesis, trafficking and release are tightly linked with endosomes and lysosomes pathways [73].

Here we will review the advances of EV research in the field of PD, with a specific focus on glia-derived vesicles. We will first introduce glia-neuron interactions in PD, their harmful/beneficial role, the “old and new actors” surfing the scene, coupled to the therapeutic potential in harnessing astroglia-derived mechanisms to boost neuroprotection and neurorepair.

We will then describe the evidences pointing out the EVs as contributors to the progression of neurodegeneration, but taking into account their potential when proposed as novel biomarkers for PD. Next, we will report the findings about the role of EVs as mediators of brain protection and repair, together with the most recent development of their application as new nanotherapeutics.

## 2. A Dual Role for Glia in Parkinson’s Disease

### 2.1. The Bad Guys

A key aspect of PD pathophysiology is neuroinflammation in the SNpc, where reactive astrocytes and microglia intersect the key cellular functions affected in PD, namely, oxidative stress and the inflammatory response, endoplasmic reticulum (ER) stress, mitochondrial, lysosomal, proteosomal and autophagic functions, converging to α-Syn aggregation and prion-like cell-to-cell transmission of α-Syn [25,74,75,76,77].

While the question of whether neuroinflammation initiates DAergic neurodegeneration is not clear at present, a dysfunction of the astroglial cell compartment is recognized to play a prominent role, in which both astrocyte and microglia activation perform significant functions [44,67,75,76,77].

It is believed that during the early degeneration process, the release of aggregated α-Syn [77] from the dysfunctional DAergic neurons, may activate glial cells to release a vast panel of pro-inflammatory factors in the SNpc microenvironment, leading to a further exacerbation of microglia and resulting in neuronal cell death [43,58,60,77,78,79]. In turn, glial activation may contribute to the overall degeneration process via a prion-like behavior of misfolded α-Syn propagation [80]. Importantly, abnormal deposition of α-Syn contributes to the pathogenesis of PD, as aggregated forms of α-Syn represent a major component of LB, and the typical feature of the pathology [25].

Of special interest, reactive astrocytes effectively phagocytose dead cells and protein aggregates like α-Syn in synapses [81], and the presence of aggregated α-Syn in astrocytes and oligodendrocytes in PD has been described [82,83]. While the mechanisms responsible for the transfer of secreted α-Syn from neurons to astrocytes and their neuro-inflammatory responses remain unclear, the two phenomena are recognized to play a major role in the progressive DArgic neuron death of PD, and other NDs [83,84].

Reportedly, aging represents a critical vulnerability factor, as with age, the increased inflammation and oxidative stress may predispose to mitochondrial dysfunction and dysregulation of lysosomal, proteasomal and autophagic functions [35,49,50,85,86,87,88,89,90,91,92]. Additionally, with advancing age, the nigrostriatal DAergic neurons progressively decline and their “adaptive” capacity is gradually reduced, leading to the late appearance of the clinical signs of PD [85,91,92,93,94,95,96,97]. An increasing body of earlier and more recent evidences suggests a prominent role of astrocytes and microglia as main players in mediating the harmful effects of aging, interacting with a specific genetic background and different environmental factors to counteract nigrostriatal self-repair.

In fact, oxidative stress and a low level of inflammation characterize the aging process and this condition is further upregulated in neurodegenerative pathologies, such as basal ganglia injury, or upon exposure to different neurotoxins, like 1-methyl-4-phenyl-1,2,3,6-tetrahydropyridine (MPTP) or 6-hydroxydopamine (6-OHDA), male gender and PD genetic mutations. Together, with age, microglial cells shift to a so-called “primed” (M1) phenotype [90,98,99], characterized by a greater production of a number of pro-inflammatory molecules in response to immune or neurotoxic challenges with detrimental effects for the vulnerable DAergic neurons. Within the harmful mediators, nuclear factor κB (NF-κB), a primary signal for inflammasome induction [79,90], associated with the pro-inflammatory cytokines, tumor necrosis factor α (TNF-α), interleukin 1β (IL-1β) and IL-6 [100]. Additionally, the generation of reactive oxygen (ROS) and nitrogen species (RNS), in turn amplify microglial activation, and engender a vicious cycle of oxidative stress and inflammation, resulting in increased neuronal vulnerability and/or death [9,29,42,44,47,49,56,59,100,101].

**Figure 1:**
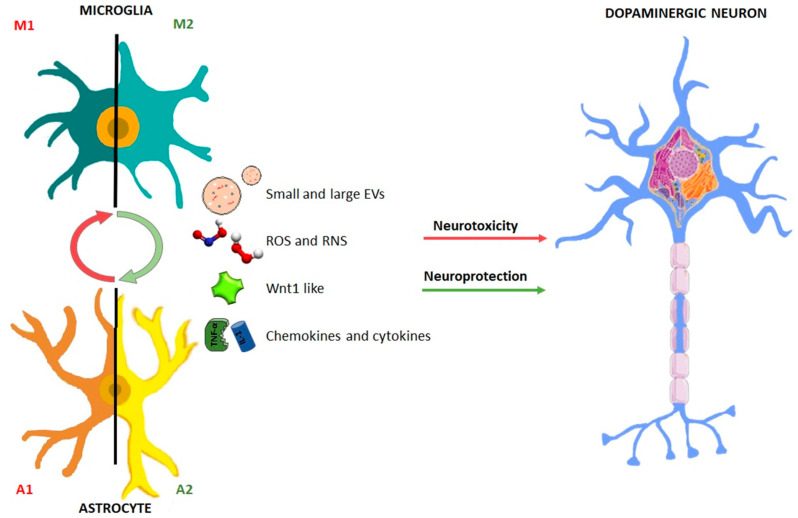
Dual role of glial cells in the regulation of the balance between neurotoxicity and neuroprotection in Parkinson’s disease. Astrocyte and microglial schematic drawing illustrating the dual facet of glial cells. Upon basal ganglia injury, glial cells acquire an activated “harmful” so-called astrocyte (A1) and microglial (M1) phenotype, characterized by up-regulated release of a number of pro-inflammatory mediators, including cytokines and chemokines, associated by generation of elevated levels of reactive oxygen (ROS) and nitrogen species (RNS), in turn generating a vicious cycle of inflammation and dopaminergic degeneration. Glial cells are also endowed with neuroprotective factors, including Wnt1, that incite neuroprotection and repair of the dysfunctional neurons. The potential glia-glia and glia-neuron crosstalk via small and large vesicles is illustrated (for detail, see the text).

Notably, such feedforward cycle of chronic glia activation and continuous damage of DAergic neurons are likely to play a decisive role for the severity of the lesion and the overall detrimental effects upon SNpc neurons, including their capacity for neurorescue/neurorepair [9,47,48,49,50]. Within this frame, astrocytes may play a dual “harmful/beneficial” role, as they can either cooperate with microglia to exacerbate M1 phenotype and enhance neurotoxicity, or they can downregulate microglia activation and promote neuroprotection and neurorepair [8]. Yet, the factors determining whether astrocytes will assume a beneficial or harmful phenotype are actively investigated. Especially, the specific way whereby astrocytes and microglia may convey the harmful/beneficial cargoes to the dysfunctional DAergic neurons remains poorly understood (Figure 1).

### 2.2. The Good Guys: Old and Novel Actors

Both astrocytes and microglia are endowed with multiple neuroprotective and pro-neurogenic properties as they release growth, neurotrophic and anti-oxidant factors, they clean-up the neuronal synapses from glutamate, they harbor receptors for endogenous anti-inflammatory molecules, and activate neurorepair and neurogenesis programs via the expression of neurogenic factors. Especially, astrocytes harbor a powerful arsenal of neurotrophic molecules and express receptors for neurotransmitters, cytokines, chemokines and hormones in cooperation with those produced by microglia [3,4,5,6,40,102,103,104,105]. An important aspect of astrocyte’s properties regards their region-specificity, as the nature of the factors they secrete can vary not only according to the CNS region, but also as a function of the age and sex of the host, and the type of brain lesion/injury [6,7,105,106,107]. Specifically, astrocytes of the ventral midbrain (VM-As) release important molecules for the development and survival of DAergic neurons, including glial-derived neurotrophic factor (GDNF), and basic fibroblast growth factor (bFGF), [11,40,104,108,109,110,111,112].

Notably, following moderate neuronal damage, activated astrocytes can support neuron survival and recovery of their synaptic input, also via an astrocyte inflammatory signaling through STAT3 playing a crucial role within the protective astrocyte phenotype [113,114,115,116,117,118,119]. In particular, the relationship between reactive astrocytes and microglia is bidirectional: astrocytes can modulate the extent of the inflammatory response, and microglia, in turn, can activate both neuroprotective and detrimental pathways for the neighboring neurons, according to the glial genotype and a plethora of environmental factors.

Significantly, when astrocyte dysfunction and microglia activation do persist upon 6-OHDA-induced nigrostriatal lesion in rats, the severity of SNpc neurodegeneration is accelerated, due to the inhibition of glial-dependent compensatory mechanisms of neuronal repair [117]. Notably, the M2 polarized microglia release the anti-inflammatory cytokines, IL-4 and IL-10, brain-derived neurotrophic factor (BDNF) and insulin-like growth factor-1 (IGF-1), as well as extracellular matrix proteins, such as fibronectin [29,102,119]. In addition, activated microglia utilize the C-C chemokine receptor-5 (Ccr5) signaling pathway to regulate astrocytic neurotransmitter production, which may be relevant in the context of sporadic PD [120,121].

A key feature of astrocyte neuroprotective properties is the activation of an antioxidant self-defense response. Indeed, DA oxidative metabolism represents a vulnerability factor linking both mitochondrial and lysosomal dysfunctions to PD pathogenesis [44,89,92,104,122]. Here, astrocytes play a critical role via the expression and up-regulation of NF-E2-related factor 2 (Nrf2), which translocates to the nucleus and binds to antioxidant responsive elements (ARE) [123,124].

Notably, oxidative stress can up-regulate the rate-limiting enzyme in GSH production (i.e., glutamate cysteine ligase) and also increase the expression and membrane targeting of multidrug-resistance associated protein-1 (MRP1) export pump, thus facilitating the efflux of GSH from astrocytes. These events promote a robust protective response to the changing redox milieu [7].

Remarkably, aging-induced decline of astrocytic Nrf2 promotes an up-regulated expression of major microglial pro-inflammatory cytokines, such as TNF-α, IL1β, IL-6 and Nos2, both at striatal [124], and SNpc levels [104,125], exacerbating oxidative stress and inflammation with harmful consequences for dopaminergic neuronal survival, a condition efficiently reversed by astrocyte grafting [104], (see the next section).

### 2.3. Beneficial Astrocyte-Neuron Dialogue Promotes DAergic Neurorepair and Activates Adult Neurogenesis

In the last decade, several lines of evidence pointed to Wingless-type MMTV integration site 1 (Wnt1)/β-catenin signaling, a chief player in DAergic neurodevelopment [61,126,127,128], as an emerging pathway involved in bidirectional astrocyte-neuron crosstalk contributing to DAergic neuron survival (recently reviewed by Marchetti, 2018 [129]). Astrocytes are known to release various region specific signaling molecules, such as sonic hedgehog (Shh) and Wnts, which may interact with each other to dictate the neurogenic behavior in the adult CNS [48,105,106,126,127,128,129,130,131,132,133]. An important feature of astrocytes is their pivotal role for defining the stem cell niche, according to a regional specificity. For example, E13.5 VM-As, but not cortex (Cx) astrocytes, express Wnt1 and Wnt5a and different DA-specific transcription factors such as Pax-2, En-1, and Otx-2 and promote the differentiation of VM embryonic precursors into tyrosine-hydroxylase positive (TH+) neurons, in vitro, supporting the prominent role of VM-As as part of the neurogenic niche promoting VM-DA neurogenesis [61,64,127,128,130,133].

In the MPTP-based mouse model of basal ganglia injury, a gene expression analysis uncovered a long-lasting upregulation of a specific Wnt’s family member component (i.e., Wnt1), associated to a 2-4-fold increase of certain pro-inflammatory chemokines (i.e., CCl3, CXCl10 and CxCl11) during both nigrostriatal degeneration and self-recovery [48]. Surprisingly enough, such an increase in Wnt1 mRNA transcription was identified in VM-astrocytes derived ex vivo from MPTP-injured midbrain. Additionally, the chemokines up-regulated “in vivo”, were found to activate astrocyte expression of Wnt1, in vitro [48].

In the Wnt signaling cascade, β-catenin represents the pivotal molecule. Here, after Wnt1 binding to the Wnt receptors, Frizzleds (Fzds), β-catenin accumulates in the cytoplasm and translocates to the nucleus, where the transcription of Wnt target genes is involved in DAergic neurogenesis and neuroprotection (recently reviewed by Marchetti et al. 2020) [64]. Remarkably, histochemical and functional recovery of nigrostriatal DAergic neurons was accompanied by significant increases of Wnt receptors and β-catenin in DAergic neurons, thus suggesting a compensatory mechanism implicated in DAergic salvage [48]. We then hypothesized that such a mechanism might contribute to the recognized capacity of nigrostriatal neurons to program a compensatory response upon injury [85,93,94,134,135,136,137,138,139,140]. In fact, previous findings indicated astrocyte and microglia activation as the source of pro-inflammatory cytokines and neurotrophic factors during DAergic nigrostriatal recovery upon injury [134,135,136,137,138,139,140]. Strikingly, astrocyte-TH neuron crosstalk was observed to accompany the recovery phase of MPTP-injured neurons, which exhibited an extraordinary neurorepair/ neurorestoration [29,48,49,61,141,142,143,144]. Then, the sharp and long-lasting increase of astrocyte’s Wnt1 and microglial-derived chemokines, CCl3, CXCl10 and CxCl11, further linked reactive astrocytes and Wnt/β-catenin signaling to nigrostriatal injury and repair, uncovering astroglial Wnt1 as a novel compensatory rescue signal for mesencephalic DAergic neurons [48,61,143,144].

The chief role of astrocytes in redirecting the unfriendly VM microenvironment both inside and outside the neurogenic niches is highlighted upon nigral transplantation of adult SVZ-NSCs in the aged MPTP mouse model of PD [125]. Interestingly we found that a large part of the transplanted neural stem cells differentiated into astrocytes within the lesioned SNpc. Here, the NSC-derived and the endogenous astrocytes activated intrinsic cues instructing inciting DAergic neurorepair [125].

Hence, astrocytes by themselves, are the key elements for DAergic neurons survival, repair and regeneration. As a proof of concept, our recent work showed that by grafting VM-As within the MPTP-lesioned SN of aging mice exhibiting motor impairment, induced beneficial antioxidant and anti-inflammatory effects highlighting astrocyte-derived factors and mechanisms as the crucial keys for successful therapeutic outcomes in PD [104].

Together, these data highlight that a “beneficial” glial-neuron dialogue is a prerequisite to promote neuroprotection and activate neurogenesis in the parkinsonian brain (Figure 1). Further investigation on the modality of such glial-to-neuron transfer/delivery of key “beneficial” cargoes, will have important implications for the identification of potential therapeutic targets to incite neuroprotection/neurorepair. Here, an overview of this intricate crosstalk between neurons and glia via EVs is presented.

## 3. The Bad Side of EVs: are they the Trojan Horse of Neurodegeneration?

### 3.1. Neuron-to-Neuron EV Propagation

From an historical perspective, and in the context of neurodegenerative diseases, the EVs were initially identified as potential contributors in the propagation of misfolded protein aggregates [145,146]. Protein aggregate disorders are regarded as a continuum, with overlapping features in neurodegenerative diseases, including the α-Syn fibrils in PD [147]. Indeed, α-Syn is one of the main players in PD onset and progression, as its aggregation in toxic oligomers, may lead to the further conglomeration in fibrils and formation of LBs in DAergic neurons, with consequent neurotoxicity and neurodegeneration [148]. As said, α-Syn propagates between neurons and between neurons and glia. Amongst the ways of propagation, EV-mediated spreading of α-Syn seems to be a route often associated with a more toxic form of aggregated α-Syn and, therefore, with the effect to fasten PD spread and progression [149]. Recent studies suggest that, although α-Syn can be either secreted alone or actively carried inside EVs, only within EVs such protein tends to form toxic oligomers. Furthermore, the oligomeric α-Syn protected inside EVs is easier taken up by the recipient cells, including glial cells [149].

The first observation that α-Syn may be localized inside cellular-deriving vesicles comes from studies made by Lee and colleagues, in 2005, which demonstrated the α-Syn-vesicle entry and secretion in differentiated SH-SY5Y cells and rat primary cortical neurons [150]. Physiologically, α-Syn, in healthy neurons is able to interact (presumably through its N-terminus region) with the membranous lipidic bilayer moieties and, thus, promote membrane curvature, thereby modulating synaptic trafficking and neuronal vesicles budding [151].

The first study to link exosome-released α-Syn with PD pathogenesis comes from Emmanouilidou and colleagues, in 2010, which firstly showed that α-Syn is secreted in exosomes by SH-SY5Y cells via a calcium-dependent endosomal mechanism [152]. They engineered the cell line to express wild-type (WT) α-Syn in a Tet-off inducible system, demonstrating that conditioned medium containing exosomes from cells overexpressing WT α-Syn induces differentiated SH-SY5Y and primary cortical neurons, used as target cells, to undergo apoptosis [152].

Notably, agents such as manganese (Mn), recognized as an environmental factor in PD-like disorders, can impact on neuronal EVs secretion [153]. Hence, in the work of Harischandra et al., in 2017 [153], the authors used MN9D DAergic cells stably expressing α-Syn, to analyze extracellularly secreted exosomes under the exposure to Mn. They found that Mn exposure for 24h induced the release of exosomes into the extracellular media prior to cell death. Of special interest, Mn treatment in α-Syn-expressing cells increased the protein Rab27a, capable to regulate the release of exosomes [153]. Furthermore, looking at small RNAs in exosomes isolated from Mn-exposed MN9D cells, a micro-RNA (miRNA) profiling analysis unveiled the expression of certain miRNAs recognized to crucially contribute to key biological pathways that are dysfunctional in PD, namely, protein aggregation, autophagy, inflammation and hypoxia [153]. As suggested by the authors, these results implicate a role of an exogenous environmental factor, Mn, in modulating extracellular miRNA content via the release of exosomes from DAergic cells, with potential implications for the progressive neurodegenerative process [153].

Altogether, the reported studies support the idea that pathologic oligomers of α-Syn carried by neuronally-derived EVs may not only spread the neurodegenerative process, in a neuron-to-neuron basis, but that also the quality and quantity of such EV cargoes are directly impacted by external environmental risk factors for PD.

### 3.2. Glia-to-Neuron EV Propagation

Additionally, glial cells are endowed with an EV machinery capable to deliver a panel of potential harmful cargoes. As expected, other studies confirmed that α-Syn can spread the disease also from neurons towards other target cells, including astrocytes and microglial cells [154,155]. In both cases, the endocytosis of α-Syn-vesicles has been found associated with the activation of a pro-inflammatory response in the recipient glial cells [154,155].

On this line, Chang et al., in 2013 [156], demonstrated that mouse BV-2 microglial cells treated with oligomeric toxic α-Syn are activated and, in turn, secrete a higher amount of inflammatory-exosomes containing high level of MHC class II (MHCII) molecules and TNF-α. Moreover, the so-produced BV-2-derived EVs, containing MHCII and TNF-α pro-inflammatory factors, were reported to induce high levels of apoptosis in rat cortical neurons [156].

Additionally, age might be a factor that strongly influences the way by which pathogenic α-Syn containing-exosomes are actively up-taken by surrounding glial cells, in particular microglia. Bliederhaeuser and colleagues, in 2016 [157], demonstrated that microglia from elderly mice is less able to uptake exosomes carrying pathogenic α-Syn, than the corresponding microglia from youngest mice. This implies that in elderly mice a higher amount of this type of EVs containing α-Syn oligomers is available in the intercellular milieu, maximizing their spread between neurons, with potentially harmful neuronal effects [157]. In light of the exacerbated activation of aged microglia releasing higher amounts of proinflammatory cytokines including TNFα secretion [29,47,49,157], it seems plausible that these microglial EVs may not only spread the disease but also deliver potent neurotoxic pro-inflammatory mediators contributing to DAergic neurodegeneration [158].

Very recently in 2019, Xia and collaborators [159] shed more light on the pivotal role played by microglia as modulator of transmission of exosome containing α-Syn. The group found that exosomes derived from PD patients’ plasma (and containing pathogenic α-Syn oligomers) preferentially target microglia in vivo, rather than neurons or astrocytes. These EVs induce microglial pro-inflammatory activation and, as a consequence, they significantly increase cellular proliferation, nitric oxide and cytokine secretion [159]. Specifically, α-Syn propagated by exosomes determines the inhibition of microglial autophagy mechanisms and the concurrent intracellular α-Syn accumulation. In turn, SH-SY5Y cells exposed to exosomes secreted by activated BV-2 cells, start to express significantly higher levels of the aggregated form of α-Syn intracellularly. This means that BV-2 cells may internalize the α-Syn contained in EVs and, additionally, they might secrete α-Syn via EVs that are, in turn, efficiently transmitted to recipient SH-SY5Y cells [159].

In summary, these results highlight the concept that EV-mediated transfer of pathologic α-Syn aggregates might function as a potential Trojan horse in PD. On one side, the α-Syn-EVs may propagate between adjacent neurons thus contributing to DA neurodegeneration spreading in space and time. On the other side, the α-Syn-EVs may target glial cells. In the case of microglial exposure to α-Syn-EVs, this may result in the exacerbation of the M1 phenotype, leading to a further spread EVs containing pro-inflammatory cytokines and aggregated, as well as toxic form of oligomeric α-Syn, to the surrounding glial and neuronal milieu, promoting a feedforward cycle of neuroinflammation-dependent degeneration.

Notably, toxic α-Syn aggregates and cytokines are not the exclusive kind of payloads carried by EVs during PD pathogenesis. In fact, a key role seems to be played also by miRNAs, as recently reviewed by our group [160]. Importantly, recent data suggest that miRNAs can be actively transferred among cells via EV-mediated shuttle and they can contribute to PD progression in the damaged brain. Winkler and colleagues in 2014 [161] demonstrated that exosomes can transport let-7, already identified as a miRNA overexpressed in PD animal models. Importantly, let-7 can activate the toll-like receptor 7 (TLR7) in neurons, leading to neurodegeneration [161,162]. As far as astrocyte-derived EVs are concerned, in the study of Datta-Chaudhuri and collaborators in 2018 [163], the stimulation of primary cortical rat astrocytes with the pro-inflammatory cytokines, IL-1β or TNFα, induced the secretion of EVs enriched of two miRNAs, miR-125a-5p and miR-16-5p. Furthermore, the treatment of primary hippocampal and cortical neurons with the astrocytes-derived EVs downregulated the expression of neurotrophin receptor K3 (NTRK3) and its downstream effector Bcl-2, via the EV-transferred miRNAs [163]. The downregulation of these neuronal targets delays dendritic growth in developing neurons, reduces dendritic complexity in mature neurons, and decreases neuronal excitability [163].

**Figure 2:**
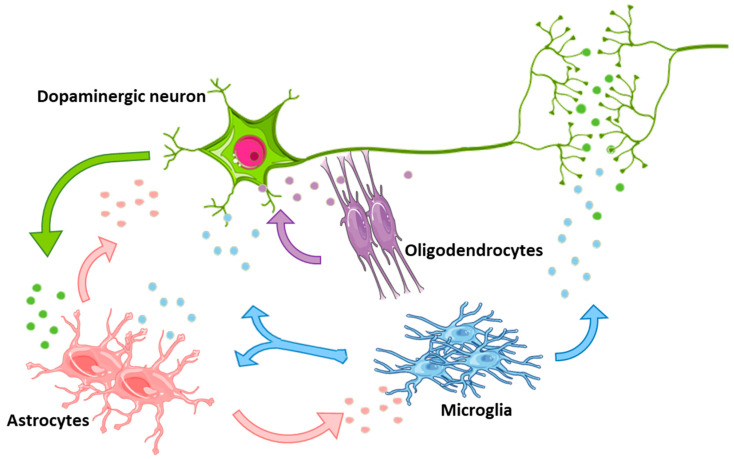
Schematic representation of glial cells and neurons communicating with each other by secretion of extracellular vesicles (EVs). Depending on the microenvironmental conditions these vesicles may mediate either harmful or neuroprotective effects. The colors of EVs reflect the donor cell type: green for neurons, light blue for microglia, pink for astrocytes and violet for oligodendrocytes.

These results strongly support the notion that, depending on the specific microenvironment (i.e., up vs. down modulation of a pro-inflammatory background), astrocytes may respond with an A1 (harmful) vs. A2 (beneficial) phenotype (discussed in Section 2). Accordingly, a differential secretion of glial-derived EVs, enriched with detrimental and/or neuroprotective and proregenerative mRNA/miRNA cargoes, are very likely to direct towards neuron death or survival [29,61,64,104,125,164,165,166] (Figure 2 and Table 1).

## 4. The Good Side of Glial-EVs: Can They be “Repurposed” as Protective for CNS?

**Figure 3:**
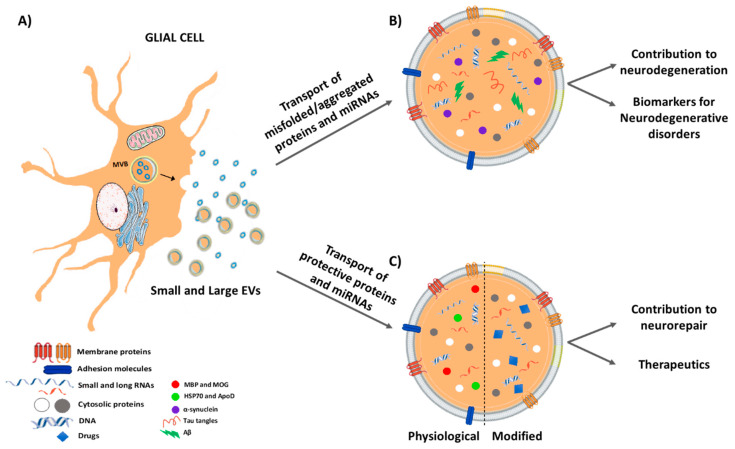
Contribution of extracellular vesicles secreted by glial cells in glia-neuron crosstalk; (**A**) Schematic representation of a glial cell releasing EVs, both exosomes (via fusion of MVBs with the plasma membrane) and shedding vesicles (directly released outside the cell). (**B**) In neurodegenerative diseases, EVs have been seen transporting unfolded/aggregated proteins or miRNAs contributing to spread the pathology. These “pathological” EVs may be used as specific biomarkers of disease. (**C**) On the contrary, physiological or modified EVs may transport protective biomolecules and may be used as therapeutic tools in neurodegenerative conditions.

As reviewed above, bidirectional communications between glial and neuronal cells subserve a variety of important homeostatic functions according to the region-specific and physio-pathological context, and as a function of a genetic and environmental background [8,9,26,29,38,49,168]. Under inflammatory, neurotoxic/neurodegenerative challenges, glia can turn into a very harmful phenotype leading to increased DAergic neuron vulnerability and/or the exacerbation of neuronal death [8,9,26,29]. However, of special importance, glial cells may also exert a panel of neuroprotective functions against DAergic neuron death, both in vivo and in vitro, (see Section 2), raising the question of whether EVs secreted by glia may crucially contribute in directing DAergic neuroprotection vs. degeneration (Figure 3A).

### 4.1. Microglial-to-Neuron EV Beneficial Effects

In a pioneering study from Potolicchio and colleagues in 2005 [169], the composition of exosomes produced by N9 microglial cells was firstly characterized. The authors found that such exosomes were enriched in CD13, an endopeptidase able to cleave neuropeptides and therefore mediating their catabolism within far regions of the CNS [169]. Other enzymes found enriched in microglial-derived EVs may have a potential therapeutic application, as demonstrated by Tamboli et al. in an Alzheimer’s disease (AD) mice model [170]. This report supports the dual facet of glia that, depending on the stimuli, may secrete EVs with beneficial therapeutic outcome.

How can pro- vs. anti-inflammatory microglial activation be alternatively selected during homeostasis or pathology? Answers came from aging mouse models. In the context of AD, Udeochu and colleagues in 2018 [171] were able to demonstrate in vivo the key importance of the exosome machinery during microglial inflammatory response. In fact, both activated and aged microglia secrete exosomes that, in turn, are able to exert anti-inflammatory mechanisms to restore the immune homeostasis. In particular, the authors found that different inflammatory mediators (e.g., immune proteins and miRNAs involved in interferon signaling and toll like receptor signaling) are secreted away from the microglia, and therefore the cells can reduce the inner pro-inflammatory status [171].

In the in vitro SH-SY5Y cell model of PD, Li and coworkers, in 2019 [167], observed the effects of EVs derived from microglial cells when administered to differentiated SH-SY5Y cells treated with the neurotoxin MPP^+^. The authors found that, while EVs derived from microglia exposed to aggregated α-Syn leads to enhanced neurotoxicity, in contrast, EVs isolated from microglia treated with unaggregated α-Syn attenuated the neurotoxicity, thereby pointing to a dual facet of microglial-derived EVs onto neuronal cells [167]. These results further support the “leitmotiv” of our earlier [8,9,38,39,40,47,48,49,50] and most recent [29,61,64,104,125,143] works underscoring that glia, depending on a panel of factors including the nature of the noxious stimulus, may exert both beneficial and detrimental effect on DAergic neurons.

### 4.2. Oligodendrocyte-to-Neuron EV Beneficial Effects

Notably, bidirectional communication between oligodendrocytes (oligo), the glial cells that synthesize the CNS myelin sheath, and neuronal cells are well recognized, with a good side of oligo-EVs being also reported [172,173,174]. Hence, in 2007, Krämer-Albers et al. [172], demonstrated that activating calcium (Ca^2+^) influx in oligodendrocytes with the calcium-ionophore ionomycin, promoted the secretion of exosomes, indicating a Ca^2+^-dependent regulation of oligo-EVs secretion. Additionally, such oligo-derived exosomes were enriched in myelin basic protein (MBP), myelin oligodendrocyte glycoprotein (MOG) and several other stress-protective proteins involved in glia-mediated trophic support to neuronal axons [172]. Interestingly, Ca^2+^ entry also mediates exosome secretion after oligodendrocyte stimulation with the excitatory neurotransmitter glutamate [173]. Especially, oligodendroglia-derived EVs have multiple effects on neurons: they modulate neuronal outgrowth and response to stress stimuli, improving neuronal viability and exerting neuroprotective effects [173]. The same group demonstrated lately that oligo-derived exosomes promote neuronal survival during oxygen–glucose deprivation, a model of cerebral ischemia. These exosomes were able to transfer to neurons both superoxide dismutase and catalase, enzymes which helped neurons to resist against oxidative stress [174].

### 4.3. Astrocyte-to-Neuron EV Beneficial Effects

Regarding astrocytes, accumulating evidence clearly suggest the ability of astrocyte-derived EVs to perform significant neuroprotective functions when challenged with noxious stimuli. Hence, earlier studies of Taylor and colleagues in 2007 [175], demonstrated that astrocytes exposed to hypothermic stimuli can secrete EVs enriched with Hsp70 which protects neurons from stress and blocks their cell death [175]. Accordingly, Wang et al., in 2011 [176], reported the ability of “stressed” astrocytes to secrete EVs enriched in glycoproteins, such as synapsin I, which, in turn, promote neuronal survival [176].

More recently, in the study of Maestro and collaborators in 2019 [177], it was found that non-stimulated astrocytes are able to secrete exosomes enriched with apolipoprotein D (ApoD), which can be internalized by SH-SY5Y cells treated with paraquat, an herbicide implicated as a risk factor in PD, used as a neurotoxic oxidative stress challenge in this cellular model of PD. In turn, this uptake mediates neuronal survival. The authors suggested that ApoD-loaded exosomes might be further characterized in vivo to treat neurodegenerative diseases, and PD in particular [177].

Of special interest, the same year Venturini and coworkers showed the ability of astrocyte processes prepared from adult rat cerebral cortex (CX), to release exosomes, and to target cortical neurons in an ex vivo/in vitro model of neuron-astrocyte co-culture paradigm. Here, astrocyte-derived exosomes were shown to be positive for the neuroprotective protein neuroglobin, thereby suggesting a feasible astrocyte-dependent neuroprotective mechanism for cortical neuron maintenance/survival [164]. The potential role of astrocyte in mediating neuroprotection is further shown by Shakespear and colleagues in their very recent work. In 2020 they demonstrated that exosomes from untreated astrocytes significantly attenuate MPP^+^-induced cell death in SH-SY5Y cells and primary mesencephalic DAergic neurons. This neuroprotective effect was lost upon treatment of astrocyte with MPP+. EVs were able to induce a reduction of mitogen-activated protein kinase4 (MKK4)—an important upstream kinase in the c-Jun N-terminal kinase cell death pathway—in target cells. Additionally, the authors described the miR-200a-3p within exosomes as the most down-regulated miRNA in MPP^+^-treated astrocytes. The treatment with miR-200a-3p mimic suppressed MKK4 expression at mRNA and protein levels, thus reducing cell death in MPP^+^-treated SH-SY5Y cells and glutamate-treated hippocampal neuron cultures. These data support the neuroprotective role of exosomal miR-200a-3p secreted by normal astrocytes, via down-regulation of MKK4 [178].

Studies from our group on the characterization of astrocyte-derived EVs from two principal brain areas affected in PD (i.e., the VM and the striatum) showed an enrichment of vesicles in the size range of small EVs (~100 nm) [179]. In addition, several positive (e.g., CD63, CD9, Tsg101) and negative (e.g., Calnexin, Tom20) EV markers have been analyzed by western blotting and confirmed the enrichment of small EVs in the astrocyte-derived vesicles of both regions [179]. Interestingly, we found that the basal EV secretion rate is specific for each brain area, with the VM releasing more EVs than the striatum. These data suggest nigrostriatal-specific differences of astrocyte-derived EVs secretion, with potential functional implications. Accordingly, Ccl3, the chemokine found to up-regulate astrocyte neuroprotective and pro-regenerative capacities (see Section 2), affects EV secretion in a region-dependent fashion, in absence of any influence dependent on cellular viability and/or proliferation differences [179]. From the identification of mRNAs and miRNAs in astrocyte-derived EVs, associated to the evaluation of their impact on DAergic neuroprotection and NSC neuronal differentiation, it seems tempting to suggest the involvement of EVs in astrocyte-neuron and astrocyte-NSC communications, in a region-specific way (Leggio et al. Ms in preparation). The underpinning of this crosstalk via EVs will be instrumental for the identification of novel mRNA/miRNA cargoes promoting neuroprotection and neurorepair in PD.

Collectively, the described studies clearly document that glial-derived EVs are capable to exert powerful detrimental or neuroprotective effects, according to different physiopathological conditions (Table 2). Intuitively, the most challenging questions still regard when, where and how the beneficial vs. harmful glial-derived EVs are differentially selected in different physiopathological conditions, and more importantly, to develop innovative EV therapies to boost neuroprotection and neurorepair in PD.

## 5. ‘Flipping the Table’: EVs as Potential Novel PD Biomarkers

PD is a neurological disorder characterized by the progressive and slow nigrostriatal DAergic degeneration, leading to a very late diagnosis (occurring when almost 60 to 70% of the nigrostriatal neurons are already lost); therefore, a major goal regards the identification of early indicators of the disease.

In general, EVs emerge as crucial players in both brain homeostasis and pathology. The identification of their content, especially in pathological conditions, including neurodegenerative diseases, might give to researchers more hints on the activated molecular pathways in the brain, as well as help the clinicians for diagnostic and prognostic purposes [180] (Figure 3B).

In the context of PD diagnosis, exploiting the characterization of detrimental vs. beneficial molecules delivered via EVs is like “flipping the table”, which may contribute to the identification of previously neglected vesicular biomarkers with a potential role also in the therapy of PD. Hence, EVs as transporters of PD biomarkers have been isolated from plasma and serum, but also from other specimens, including cerebrospinal fluid (CSF) and urine [160].

Protein and other molecules, including miRNAs, carried by EVs produced by neurons and glial cells might be used as PD biomarkers in the near future. In fact, given their lipidic nature, EVs represent ideal carriers of potential PD biomarker, able to transport their payload from the CNS, through the BBB, finally arriving to the systemic circulation. Importantly, EVs in the blood are stable for long time and they may be detected with the use of a non-invasive blood sampling [181].

For example, the above-mentioned let-7 family of miRNAs—found in blood-derived EVs of PD mouse models—is an important modulator of neuroinflammatory processes, and often found upregulated in PD microglial cells [182,183]. Additionally, detrimental aggregates of α-Syn, contained in EVs, may be used as PD biomarker, as they are higher in PD subjects compared with controls. Importantly, it has to be taken into account that the outcome depends from the source of the EVs carrying α-Syn, which can be plasma, serum or CSF. In fact, α-Syn detected in plasma deriving EVs may be also be produced by plasma cells [184,185].

With the goal of characterizing the specific composition of brain-derived EVs, Chiasserini et al., in 2014 [186], setup a proteomic analysis of CSF-derived vesicles. The EV-proteome resulted enriched in in exosome markers such as alix and syntenin-1, heat shock proteins and tetraspanins. Interestingly, the authors could identify not only neuron-specific proteins (e.g., enolase 2, dihydropyrimidinase-related protein 2, vesicle-associated membrane protein 2), but also glial specific markers (e.g., microglial integrin alpha-M and receptor-type tyrosine-protein phosphatase or glial derived nexin). The study underlines that also the fraction of EVs originating by glial cells might have an important role in the intricated CNS intercellular crosstalk both in physiology and in pathology [186].

Notably, peripheral EVs may also impact PD physiopathology both with beneficial and harmful effects. In fact, Tomlinson and colleagues, in 2015, [187] isolated EVs from PD patients’ serum. They further administrated the isolated EVs to rat cortical neurons with induced neuronal stress, observing that the EV treatment had significant protective effects in neurons. The authors suggested that a further immunophenotyping of circulating exosomal subpopulations in PD may lead to better understanding the systemic response to neurodegeneration and finally help the development of novel therapeutics [187].

On the other hand, Jiang and colleagues in 2019 [188] linked serum EVs from PD patients to DAergic neurodegeneration. They found that EVs from PD patients’ serum contained high level of miR-137, which negatively regulates oxidation resistance 1 (OXR1) gene expression, inducing oxidative stress in DAergic neurons and thus triggering cell death [188]. Here, the effects of serum-derived exosomes were investigated both in rodents and in cellular models of PD, after exposure to oxidative stress injury. Using loss-of-function experiments with miR-137 antagomir, it was found that inhibition of exosomal miR-137 ameliorated PD-induced oxidative stress injury in vitro and in vivo, which alleviated oxidative stress and via up-regulation of OXR1 [188].

Together, serum EVs from PD patients were shown to harbor both neuroprotective and neurotoxic cargoes, thus posing a number of challenging questions, including the central vs. peripheral origin of serum EVs.

Within this context, in order to overcome the issue of specific EV-donor-cell identification, and to allow the distinction between neuron-, astrocyte- and oligodendrocyte-derived EVs, once isolated from plasma of PD patients, Ohmichi and colleagues in 2018 [189] generated an ELISA-based platform. The platform was able to successfully discriminate the three possible EV donors within the CNS, based on their specific expression of the common small EV marker CD81, coupled with either neuron-specific SNAP25 or astrocyte-specific EAAT1 or oligodendrocyte-specific OMG markers. The authors claim that the augmented levels in patients with PD of neuron- oligodendrocytes-specific EVs may help the PD staging, and therefore potentially be useful as surrogate biomarker to follow PD progression in patients [189]. This study opens up the avenues towards specific identification of neuron- and glia-derived EVs from plasma of PD, MSA and PSP patients, for example by using immune-specific assays. These novel methodologies will help to better distinguish the dual role played by glial cells in neurogenerative diseases and, in general, during neuro-inflammation [189].

In addition to protein, from studies on PD patients’ serum or CSF-derived EVs, specific miRNAs have been found either enriched or depleted in correlation with PD onset. In particular, putting together the analyses of different sequencing-based studies, it was reported a blood-derived EVs enrichment of miR-195, miR-24, miR-153, miR-409-3p, miR-10a-5p and let-7-c-3p, miR-331-5p or a depletion of miR-19b, miR-1 and miR-505 compared with EVs from controls. As previously observed for protein payloads, it might be difficult to predict the specific CNS EV-donor cell [190,191,192]. Yet, it is very challenging to understand if systemically-derived EVs (from CSF, serum, plasma, urine etc.), carrying functional miRNAs, might originate from neurons or from glial cells. Thus, the role of glial cells in miRNA-EV mediated production and delivery, which is gaining growing importance in PD onset and development, still remain elusive. In the near future, additional research concerning cell-to-cell propagation of miRNAs between neurons and glial cells, together with more specific protocols for EV recovery and identification from biological fluids, might allow the development of novel strategies to discriminate the exact nature of EV-donor cells.

Altogether, as suggested from the studies reported above, in the Era of personalized medicine, further research aimed at a more precise identification of the specific cellular origin of the EVs, might improve not only diagnosis and prognosis, but also help to precisely direct the therapy, in patients with CNS conditions, including PD.

## 6. Maximizing the Potential: EVs as Next-Generation of PD Nanotherapeutics

Whether it is possible to boost EV beneficial potential is the next logical question. EVs bear the great advantage of being biocompatible carriers, able to increase the bioavailability of their cargoes and to easily cross the BBB, therefore delivering their cargoes within the CNS. This feature is of pivotal importance in the design of drugs directed to treat CNS pathologies, including neurodegenerative diseases, where the drug molecular targets may be located far from the systemic circulation, on the other side of the BBB, inside the brain [193] (Figure 3C). This is particularly relevant for PD, currently not curable, but with palliative treatments available, able only to slow down symptoms [194].

Dopamine (DA) is a drug often used to fight tremor and bradykinesia associated with PD. Qu and collaborators [195], in 2018, tested DA-loaded exosomes in a PD mouse model. The group demonstrated that CNS distribution of DA was significantly higher when DA is encapsulated in EVs and with lower associated systemic toxicity [195]. Another way to treat PD consists in eliminating toxic α-Syn aggregates. To pursue that, Cooper and colleagues, in 2014, [196] peripherally injected in PD mice exosomes loaded with siRNA against α-Syn. Seven days after injection, the authors found significant reduction in intraneuronal α-Syn aggregates, including in DA neurons of the SNpc [196]. Moreover, Izco and colleagues, in 2019 [197], efficiently delivered shRNA minicircles silencing α-Syn, carried by exosomes, administered systemically to PD mice, with the effects of reducing brain α-Syn aggregation and diminishing DA neuronal loss, with improved PD symptoms [197].

EVs may be “re-programmed” to express beneficial molecules able, in turn, to promote neurorepair and block neuroinflammation. This can be pursued by using producer cells other than neurons or glia, such as macrophages or stem cells. Ultimately, these cells are able to produce and package into EVs different kind of active biomolecules such as miRNAs, mRNA, proteins directed to ameliorate PD symptoms or block its development [198]. For example, Haney and colleagues, in 2013, [199] systemically administered to PD mice macrophages overexpressing catalase resulting in reduction of inflammation and neuroprotection [199]. The following year, the same group systemically administered to PD mice macrophages overexpressing glial cell-line derived neurotropic factor (GDNF), with the analogue result of ameliorated neurodegeneration and neuroinflammation [200].

Several groups demonstrated that stem cells derived from the dental pulp have unique neurogenic proprieties. Jarmalavičiūtė and collaborators, in 2015, [201] demonstrated that exosomes from dental pulp stem cells rescue human DA neurons from 6-OHDA-induced apoptosis [201]. The same group, in 2019, demonstrated that the same stem cells-derived exosomes may work also in vivo in a mouse PD model, when administered intranasally. The observed effects were improvements of motor symptoms and normalization of TH expression in both SNpc and striatum [202]. In the same direction, Kojima and colleagues [203], in 2018, designed intracerebrally implanted human mesenchymal stem cells, able to deliver into PD mice brain exosomes enriched with therapeutic catalase mRNA, which, once delivered and translated in target cells, attenuates neurotoxicity and neuroinflammation [203].

Altogether, the reported results, although still in preclinical models, are very encouraging. In particular, it was observed that glial cells have a key role in driving their activation alternatively towards a pro-inflammatory or an anti-inflammatory phenotype and this have profound effects on the overall neuronal survival during the progression of PD. Especially, the unique ability of astrocytes, by themselves, to fulfill the role of neuroprotectant, has been recently highlighted by grafting VM-As, “in situ” in the aged SN, which results in a robust neuroprotection against MPTP-induced nigrostriatal toxicity, and is mediated by a “beneficial” glial-to-neuron crosstalk via an Nrf2-driven Wnt/β-catenin pro-survival Axis [104]. It will be of great importance to understand whether the protective crosstalk between astrocytes and neurons is mediated by astrocytes-released EVs and therefore characterize glia-EVs content and functional impact(s). This information will open-up novel scenarios, implementing the characterization of glial cells as potential producers of a novel generation of PD-nanotherapeutics.

## 7. Conclusions

PD is a multifactorial disease, where a complex interplay between several genes and many environmental factors, especially aging, oxidative stress and inflammation, contribute to the demise of nigrostriatal DAergic neurons, with causes and mechanisms not clearly defined. Actually, there are no effective treatments that can stop or reverse the neurodegeneration process and current treatments rely on DAergic drugs, which only temporarily alleviate motor symptoms.

Here we focused on the pivotal role of glial cells in the parkinsonian brain, as critical sources of “harmful” and “beneficial” mediators, promoting either detrimental or neuroprotective effects onto their neuronal counterparts.

Within this intense glia-to-neuron dialogue, a privileged way of sending and receiving messenger molecules is through EVs-mediated signaling. For their nature, EVs are membranous bilayers, able to store and deliver biomolecules, protected from the extracellular milieu and to reach far sites, even to cross the BBB. As anticipated, a most challenging question still regards how the beneficial vs. the harmful glial-derived molecules can be delivered to the vulnerable DAergic neurons, and if the glial EV machinery has a principal role in such a complex neuron-glia crosstalk.

We know that EVs are involved in the storage and delivery of pathologic oligomers of α-Syn and other pro-inflammatory mediators, which may spread the neurodegenerative process, together with the exacerbation of the proinflammatory phenotype. More importantly, external environmental risk factors for PD can directly modulate both the quality and quantity of such EV-cargoes, thereby functioning as a potential Trojan horse for the vulnerable PD neurons.

Because EVs can leave the brain and reach the blood circulation, this feature makes them potential transporters of PD biomarkers. Hence, the characterization of EV-derived molecular messengers during PD onset, may help to diagnose the pathology at an earlier stage, when it might be possible to delay and/or stop the neurodegenerative process.

One key point in the future will be to distinguish amongst the specific sub-groups of EVs, depending on their specific cellular origin (neuronal vs. glial). In PD and in other neurodegenerative diseases, this characterization will help to better identify the molecular mechanisms activated, but also to personalize the diagnosis. Once feasible, this will help to better stratify the PD patients, based on the specific ongoing crosstalk between glia and neurons.

On the other hand, glial-derived EVs can also perform significant neuroprotective functions as documented by an increasing number of studies. Importantly, “stressed” astrocytes challenged with different stimuli, secrete EVs enriched with neuroprotective factors, in turn, promoting neuronal survival.

Clearly, the identification of specific cargoes in astrocyte-derived EVs will be fundamental to further extend our knowledge on the complex neuron-glia circuitry in PD, and may be exploited for the development of innovative EV-derived therapies. In fact, the EVs trafficking can go through the other way around, from the periphery, across the BBB, towards the CNS. Many are the preclinical examples of systemically and nasally-delivered EVs containing drugs (including dopamine or catalase) able to ameliorate PD symptoms. In the future, patient-specific engineered-EVs might be used to re-direct the harmful microenvironment and sustain DAergic recovery at earliest phases during PD onset and progression.

These new notions are at a pre-clinical stage, and further studies are needed to develop innovative EV-based therapies. However, it is foreseeable that the characterization of the exact role played by glial-derived EVs will greatly advance our understanding of neuron-glia communication. Moreover, the optimization of protocols for EV purification and modification to generate improved lipophilic therapeutic carriers bear the potential in a near future to boost neuroprotection and neurorepair in PD and for a wide number of chronic neurodegenerative diseases.

## Figures and Tables

**Table 1:** Description of the detrimental effects mediated by EVs released by neurons, microglia or astrocytes on neuronal target cells.

DONOR CELLS		TYPE OF TREATMENT	EFFECTS OF EVs	REF.
**NEURONS**	Differentiated SH-SY5Y cell line	Overexpression of WT or mutant A53T/A30P α-Syn	α-Syn propagation and aggregation; triggering of apoptosis	[150,152]
	MN9D dopaminergic cells	Stably expression of α-Syn and Manganese (Mn) exposure	Regulation of miRNAs involved in protein aggregation, autophagy, inflammation and hypoxia	[153]
**MICROGLIA**	BV-2 microglia cell line	Aggregative α-Syn exposure	High levels of MHCII molecules and membrane TNF-α; induction of neuron cells apoptosis	[156]
	Microglia from aged mice	None	Spreading of α-Syn oligomers and pro-inflammatory factors	[157,158]
	BV-2 microglia cell line	Exosome from PD patients’ plasma	Exacerbation of pro-inflammatory phenotype; spreading and aggregation of α-Syn	[159]
	BV-2 microglia cell line	Aggregated α-Syn	Enhanced MPP+ induced neurotoxicity in neuronal cells	[167]
**ASTROCYTES**	Primary cortical rat astrocytes	Pro-inflammatory cytokines (IL-1β or TNFα)	Transport of miR-125a-5p and miR-16-5p; downregulation of NTRK3 and Bcl-2 in hippocampal and cortical neurons	[163]

**Table 2:** Description of beneficial effects mediated by EVs released by microglia, oligodendrocytes or astrocytes on neuronal target cells.

DONOR CELLS		TYPE OF TREATMENT	EFFECTS OF EVs	REF.
**MICROGLIA**	BV-2 microglia cell line	Statins exposure	Enrichment in IDE enzyme and enhanced amyloid β-peptide clearance	[170]
	BV-2 microglia cell line	INFγ stimulation	Transport of immune proteins and miRNAs, such as miR-155 involved in the resolution of interferon mediated activation	[171]
	BV-2 microglia cell line	Unaggregated α-Syn	Attenuated MPP+ induced neurotoxicity in neuronal cells	[167]
**OLIGODENDROCYTES**	Primary oligodendrocytes	Calcium-ionophore ionomycin	Enrichment in MBP, MOG and several other stress-protective proteins involved in glia-mediated trophic support to neuronal axons;	[172]
	Primary oligodendrocytes	Neurotransmitter glutamate stimulation	Modulation of neuronal outgrowth and response to stress stimuli; improvement of neuronal viability and neuroprotection	[173]
	Primary oligodendrocytes	Oxygen–glucose deprivation	Promotion of neuronal survival under oxygen-glucose deprivation; transfer of superoxide dismutase and catalase	[174]
**ASTROCYTES**	Primary astrocytes	Hypothermic stimuli exposure	Enrichment in Hsp70; enhanced neuroprotection	[175]
	Cortical astrocytes	High concentration of KCl	Enrichment in synapsin I which promote neuronal survival	[176]
	Primary astrocytes	None	Enrichment in ApoD; stimulation of neuronal survival under oxidative stress conditions	[177]
	Adult cortical astrocytes	None	Expression of neuroglobin involved in cortical neuron maintenance/survival	[164]
	Primary astrocytes	None	Enrichment in miR-200a-3p; downregulation of MKK4 and neuroprotection	[178]
